# Laser-Based Propagation of Human iPS and ES Cells Generates Reproducible Cultures with Enhanced Differentiation Potential

**DOI:** 10.1155/2012/926463

**Published:** 2012-05-30

**Authors:** Kristi A. Hohenstein Elliott, Cory Peterson, Anuradha Soundararajan, Natalia Kan, Brandon Nelson, Sean Spiering, Mark Mercola, Gary R. Bright

**Affiliations:** ^1^Intrexon Corporation, Cell Engineering Unit, 6620 Mesa Ridge Road, San Diego, CA 92121, USA; ^2^Cyntellect, Inc., 6620 Mesa Ridge Road, San Diego, CA 92121, USA; ^3^Sanford-Burnham Medical Research Institute, 10901 Torrey Pines Road, La Jolla, CA 92037, USA

## Abstract

Proper maintenance of stem cells is essential for successful utilization of ESCs/iPSCs as tools in developmental and drug discovery studies and in regenerative medicine. Standardization is critical for all future applications of stem cells and necessary to fully understand their potential. This study reports a novel approach for the efficient, consistent expansion of human ESCs and iPSCs using laser sectioning, instead of mechanical devices or enzymes, to divide cultures into defined size clumps for propagation. Laser-mediated propagation maintained the pluripotency, quality, and genetic stability of ESCs/iPSCs and led to enhanced differentiation potential. This approach removes the variability associated with ESC/iPSC propagation, significantly reduces the expertise, labor, and time associated with manual passaging techniques and provides the basis for scalable delivery of standardized ESC/iPSC lines. Adoption of standardized protocols would allow researchers to understand the role of genetics, environment, and/or procedural effects on stem cells and would ensure reproducible production of stem cell cultures for use in clinical/therapeutic applications.

## 1. Introduction

Human embryonic and induced pluripotent stem cell (ESC, iPSC) lines have been derived and maintained in a variety of ways, creating extensive variability and inconsistency from laboratory to laboratory. Currently, ESC and iPSC lines are cultured under diverse conditions, involving numerous methods of expansion, both feeder-dependent and feeder-independent matrices, and a variety of medium formulations [1–8]. Large-scale practical utilization of human ESCs and iPSCs for drug discovery applications, developmental and disease models, and regenerative therapeutic applications, will require more consistent and scalable culturing methods. Likewise, generation of GMP-quality stem cell lines will require standardized, traceable methods for stem cell derivation and expansion [9].

Manual passage, using specialized stem cell knives, razors, or pipettes to physically section stem cell colonies, is widely accepted as the best method for propagation of human ESC and iPSC lines. Manual propagation of stem cell lines does not involve the use of enzymes and therefore is thought to better maintain genetic stability of human ESCs and iPSCs in long-term culture [10–14]. Other benefits of manual expansion of stem cell cultures include passage of similar sized cell clumps, low cellular trauma, and selective transfer of specific undifferentiated colonies [7, 15]. However, scale-up of multiple stem cell lines using these methods is unattractive because of the high labor cost, inconsistency of output associated with varying expertise, risk of contamination, and the inability to effectively automate. Due to these technical demands associated with manual passage, routine propagation of most human ESC and iPSC lines is often performed using enzymatic passage [5, 6]. Enzymatic methods (e.g., using accutase, collagenase, dispase, trypsin, or TrypLE) are usually used for large-scale expansion and are well suited for automated platforms [16]. However, these methods are highly problematic as enzymatic dissociation results in variable-sized colonies leading to significant inconsistency among cultures [7]. Also, human ESCs/iPSCs do not survive well as single cells which limit the utility of enzymatic propagation [17]. While chemical compounds (e.g., ROCK inhibitor) may be used to promote survival of dissociated stem cells, these compounds do not alleviate the heterogeneity associated with enzymatically passaged human ESC and iPSC cultures, nor is the full impact of routine usage of such compounds known [18]. The large heterogeneity in colony size also limits the usefulness of high density plates, such as 96-well and 384-well plates, for higher throughput applications.

This study reports a novel approach for the expansion of human stem cell lines using laser-based propagation. Laser-mediated passaging was performed by precise cutting of human stem cell cultures by a laser into specific sized cell sections. These cell sections were transferred by simple pipetting to new culture dishes for propagation. The cell sections were of uniform size leading to greater uniformity of the resulting colonies. Additionally, enzyme-free conditions were maintained throughout and all processing occurred within a sterile, closed environment. Operating within standard format multiwell plates enable incorporation of automated robotic systems for scalable delivery of standardized stem cell cultures.

Laser-mediated propagation maintained the quality and pluripotency of ESC/iPSCs and led to enhanced differentiation potential. This approach removes the variability associated with passaging stem cells, which should greatly improve the evaluation of gene expression signatures, genetic/epigenetic profiles, and differentiation capabilities/efficiencies of stem cell lines. Laser-mediated ESC/iPSC passage significantly reduces the expertise, labor, and time associated with manual passaging techniques and provides the basis for reproducible propagation of GMP-quality human stem cell lines.

## 2. Materials and Methods

### 2.1. Human ESC and iPSC Culture

Four human iPSC lines (BIMR 6, P15–40, and BIMR 14, P25–40 generated from adult fibroblasts, BIMR A, P20–50 and BIMR L, P30–45 generated from fetal fibroblasts, all transduced with retroviruses containing *Oct4*, *Sox2*, *Klf4*, and *c-Myc* genes, Burnham Institute for Medical Research (BIMR)) were cultured in KODMEM supplemented with 20% KnockOut Serum Replacement, 1% GlutaMax, 1% nonessential amino acids, 0.1 mM 2-mercaptoethanol, and 8 ng/mL bFGF (all from Invitrogen). iPSCs were expanded on PMEF-CFs (Millipore) or Matrigel (BD Biosciences, in medium conditioned using PMEF-CFs), medium was changed every day and cells were passaged at 1 : 2–1 : 8 every 5–7 days. H9 human ESCs (P35–65) were cultured using the same medium on PMEF-CFs and were passaged 1 : 2–1 : 4 every 5–7 days. Experiments involving H9 human ESCs were performed at the BIMR Stem Cell Core.

iPSCs were passaged by several methods including manual passage using a pipette tip or using StemPro EZPassage Disposable Stem Cell Passaging Tool (Invitrogen), enzymatic passage using collagenase IV (Invitrogen) or 0.05% trypsin (Invitrogen), and laser-mediated passage using the LEAP Cell Processing Workstation (Intrexon Corp).

Stem cell colony size was determined by measuring the longest diameter of colonies from brightfield images and by manual counting of Hoechst (Invitrogen) stained nuclei from fluorescent images. Whole well brightfield images of stem cell cultures were acquired on LEAP (Intrexon Corp) and Celígo (Brooks Automation, Inc). To generate ESC/iPSC growth curves, 0.25% trypsin (Invitrogen) was used to produce a single-cell suspension of ESC/iPSCs which were then counted using a hemocytometer on days 0–5 after passage.

Transfer efficiency after laser-mediated passage was determined by manual counting of the number of ESC/iPSC sections per well after sectioning (prior to section removal by pipetting), after removal of sections from the processed well, and after transfer of sections to new culture plates using whole well brightfield images. Two days after passage, passage efficiency was determined by manual counting of the number of alkaline phosphatase positive colonies in cultures containing transferred sections.

### 2.2. Laser-Mediated Passage

Optimal laser processing conditions were established assessing laser power, laser spot size, and density of laser spots for cutting stem cell colonies into sections with minimal loss of cells. Assessment was performed empirically by testing the ability of a given condition to consistently cut typical stem cell cultures across 96-, 12-, and 6-well plates. Photothermal laser processing was chosen to minimize cell loss during processing [19]. Laser pulse powers from 3–10 *μ*J and laser spot sizes from 10–25 um were systematically evaluated for sectioning through stem cell cultures of varying thickness. It was determined that ~8 *μ*J laser power delivered in a 10 um spot size was sufficient to section cultures of all thicknesses. To create a continuous sectioning line, laser pulses were positioned ~16 *μ*m apart in a line to effectively cut stem cell colonies. After processing, samples were washed, sections were dislodged by pipetting using normal iPSC/ESC medium, and all sections were transferred to fresh culture plates containing PMEF-CFs or Matrigel. Cells were passaged at 1 : 2–1 : 8 every 5–7 days.

### 2.3. Human ESC/iPSC Differentiation

Embryoid bodies (EBs) were generated using collagenase IV treatment of day 5 human iPSC cultures for 0.5–1.0 hour to remove colonies from culture dishes. Colonies were grown in differentiation medium in suspension culture using Ultra Low Attachment plates (Corning). The longest diameter of resulting EBs was manually measured using brightfield images acquired on LEAP (Intrexon Corp) on day 4 or 5 of suspension culture.

Human iPSCs were induced to spontaneously differentiate in medium composed of KODMEM supplemented with 20% KnockOut Serum Replacement, 1% GlutaMax, 1% nonessential amino acids, and 0.1 mM 2-mercaptoethanol (Invitrogen). EBs were grown in suspension culture for 8 days and then plated onto gelatin-coated plates and allowed to differentiate for an additional 8 days. Medium was changed every other day. Cultures were fixed on day 16 for immunocytochemical analyses.

Human iPSCs were induced to form cardiomyocytes by culturing EBs for 4 days in suspension culture in medium composed of KODMEM (Invitrogen) supplemented with 20% fetal bovine serum (FBS, Hyclone), 1% Glutamax, 1% nonessential amino acids, and 0.1 nM 2-mercaptoethanol (Invitrogen). On the 4th day, EBs were plated onto gelatin-coated plates and allowed to differentiate for an additional 18 days (22 total days). Medium was changed every other day. RNA was collected for QRT-PCR analyses on day 16, the number of contracting EBs was counted on days 16 and 22, and cultures were fixed for immunocytochemical analyses on day 22.

Human iPSCs were induced to form neural rosettes by culturing EBs for 7 days in suspension culture in medium composed of 50% DMEM/F12, 50% Neurobasal medium supplemented with glutamax, 0.5x N2 supplement, 0.5x B27 supplement (Invitrogen), 0.5 mM ascorbic acid, 0.1% albumin, 4.5 × 10^−4^ M MTG (Sigma), and bFGF (20 ng/mL, Peprotech). On the 7th day, EBs were plated onto gelatin-coated plates in the medium described above supplemented with EGF (20 ng/mL, Peprotech) and allowed to differentiate for an additional 4 days. On day 11, the number of EBs containing ≥1 neural rosette was manually counted.

### 2.4. Immunocytochemistry

Cells were fixed in 4% paraformaldehyde in PBS for 15 min, permeabilized in 0.1% Triton-X100 in PBS for 5 min, and then blocked for 1 hour in blocking buffer (10% serum of the same species as the secondary antibody, 0.05% Triton X-100 in PBS). Cells were washed and incubated with primary antibodies in 1% serum (same species as the secondary antibody) in PBS for 2 hours at room temperature or overnight at 4°C. Human iPSC/ESCs were characterized using the following antibodies: Oct4, Sox2, and Nanog (R & D Systems), SSEA4 (Developmental Studies Hybridoma Bank), and TRA1-60 and TRA1-81 (Santa Cruz Biotechnology). Apoptosis was analyzed using the following antibodies: caspase-3 and cleaved PARP (BD Biosciences) with staurosporine treatment of iPSC cultures used as a control (10 *μ*M staurosporine, 4 hour treatment). Differentiated iPSCs were characterized using the following antibodies: Nestin, Map2, ANP (NPPA), and Troponin I (Millipore), Brachyury and Sox17 (R & D Systems), AFP and *α*-actinin (Sigma), and *α*-MHC (Developmental Studies Hybridoma Bank). Cells were then washed and incubated with Alexa Fluor-conjugated secondary antibodies (Invitrogen) for 2 hours. All antibodies were diluted according to manufacturer's instructions. Cell nuclei were stained with Hoechst (Invitrogen). All images were acquired using the LEAP system (Intrexon Corp).

### 2.5. Alkaline Phosphatase Staining

Cells were fixed in 4% paraformaldehyde in PBS for 15 min and stained with Fast Red TR hemi (zinc chloride) salt (Sigma) and Naphthol, AS-MX phosphate alkaline solution (Sigma) in H_2_O for 15–30 min. All images were acquired using the LEAP system (Intrexon Corp).

### 2.6. Quantitative RT-PCR Analysis

RNA was prepared using the RNeasy Micro/Mini Kit (Qiagen) and cDNA synthesis was performed using the ABI High Capacity cDNA Reverse Transcription Kit. QRT-PCR was performed in triplicate for each primer set and in each cell sample using an ABI 7900HT Sequence Detection System. Amplification was performed using the Taqman Univeral PCR Mastermix (ABI). Specific primers and probes for stem-cell-associated genes, differentiation-associated genes, and cardiomyocyte genes were obtained from ABI. Stem-cell-associated genes included *Pou5f1* (Hs00999632_g1), *Sox2* (Hs01053049_s1), *Nanog* (Hs02387400_g1), *Tert *(Hs99999022_m1), *Zfp42* (Hs00399279_m1), *Dppa2* (Hs00414515_m1), and *Esg1* (Hs00988349_g1). Cardiomyocte-associated genes included *Nkx2.5* (Hs00231763_m1), *TnnI3* (Hs00165957_m1), *Actn1* (Hs00998100_m1), *Mef2C* (Hs01554599_m1), *Myh6* (Hs00411908_m1), and *Nppa* (Hs00383230_g1). Expression levels of all genes were normalized to Eukaryotic 18s rRNA (Hs99999901_s1), and then analyzed using the 2^ΔΔCt^ method [20].

### 2.7. Karyotype Analysis

Live cell cultures were analyzed by Cell Line Genetics. Cytogenetic analysis was performed on twenty G-banded metaphase cells.

### 2.8. aCGH Analysis

Genomic DNA was collected and purified using the Gentra Puregene Cell Kit (Qiagen). Hybridization was conducted with the 44 K Human StemArray (Ambry Genetics), with a resolution of ~24 kb over the entire genome and high resolution exonic coverage in known stem-cell-associated genes, tumor suppressors, and oncogenes. Samples were hybridized to a sex-matched pooled normal reference DNA (Promega). Data was analyzed by Ambry Genetics using DNA Analytics (Agilent) and reported using genome build HG18.

### 2.9. Statistical Analysis

Statistical analyses were performed using GraphPad Prism with a *P* value of ≤0.05 considered to be significant. One way analysis of variance (ANOVA) with Bartlett's test for equal variances was performed to evaluate resulting colony sizes generated after passage by five techniques (*n* = 20 colonies/sample, Figure 2(c)) and resulting EB sizes generated from laser-mediated, collagenase, and trypsin-passaged cells (*n* = 30 EB/sample, Figure 5(b)). Statistical analysis of QRT-PCR data (*n* = 3, Figures 4(c) and 5(d)) was performed using a two-tailed *t*-test.

## 3. Results

### 3.1. Optimization of Laser-Mediated Passage

Laser-mediated passaging conditions were optimized using four human iPSC lines and one human ESC line. Human iPSC/ESC cultures were initially passaged by standard methodology (i.e., collagenase treatment plus manual scraping of cultures) into plates containing mitomycin c-treated murine embryonic fibroblasts and cultured for 5 days in iPSC/ESC medium. Laser-mediated cutting of stem cell colonies into clumps or sections of cells was facilitated by addition of a reagent to increase photothermal absorption of the laser's energy by the culture medium [19]. Laser processing conditions were optimized with respect to laser power, laser spot size, and number of laser shots required to effectively cut cultures with minimal loss of cells.

**Figure 1:**
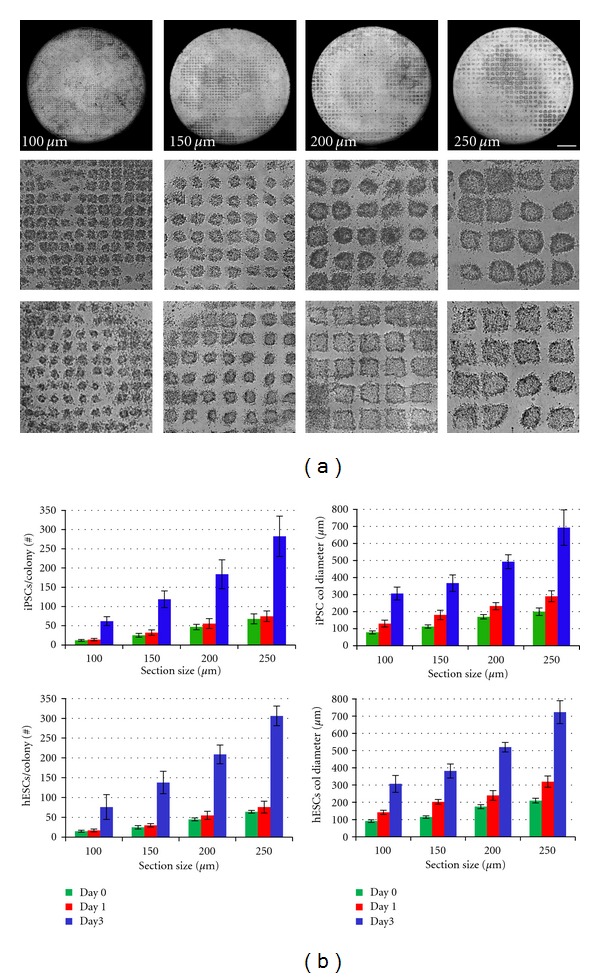
Stem cell colony size was controlled by section size using laser-mediated passage. (a) Brightfield images of human iPSC (top, middle) and ESC (bottom) cultures cut into 100–250 *μ*m sections. Scale bar, 1 mm. (b) Colony size over time following propagation of 100–250 *μ*m iPSC sections (top) or ESC sections (bottom) by laser-mediated passage. Number of cells per colony were manually counted using Hoechst stained cultures (left, *n* = 15 colonies per data point). Longest diameter of each colony was manually measured using brightfield images (right, *n* = 15 colonies per data point). Data are shown as mean + s.d.

To determine the impact of section size on resulting colony size, stem cell cultures were cut into square cell sections ranging from 75 to 300 *μ*m in size and transferred to new culture dishes by gentle pipetting (Figure 1(a), hiPSCs (top, middle), hESCs (bottom)). Sections below 75 *μ*m contained very few cells (<8 cells/section), whereas 300 *μ*m sections were too large to easily remove from the plate by gentle pipetting alone. Stem cell colony size and number of cells per colony were assessed by brightfield imaging and fluorescent staining of nuclei, respectively, after processing cultures into 100–250 *μ*m sections (Figure 1(b)). Human iPSC cultures sectioned into 100, 150, 200, and 250 *μ*m sizes resulted in sections containing 12, 25, 47, and 68 cells, respectively (Figure 1(b), top). Three days after passage human iPSC colonies measured 306, 367, 493, and 693 *μ*m in diameter with 62, 119, 184, and 283 cells per colony, respectively. Similar results were obtained with all iPSC cell lines (data not shown) and with human ESCs (Figure 1(b), bottom) using the same laser processing conditions.

For routine propagation of iPSC and ESC cultures, section sizes of 200 *μ*m were used, which allowed consistent splitting every 7 days. Notably, other groups have identified 200 *μ*m (50–100 cells) as the optimal clump size for passage [1, 5, 15, 21]. Propagation of human iPSCs using 200 *μ*m sections was highly efficient with an average transfer efficiency of 91 ± 2% with 93 ± 5% of the transferred sections forming viable colonies for an overall passage efficiency of 85 ± 3%. Passage of human ESCs resulted in similar data (overall passage efficiency of ~82%, data not shown). Laser-mediated passage with the current system required a total of ~50–90 min to process an entire plate of stem cells (depending on plate type using 200 *μ*m sections; with the majority of time (>90%) spent for laser processing and only a few minutes spent by the user). iPSCs and ESCs cultured under feeder-free conditions were also successfully propagated using the same laser-mediated passage conditions. These data demonstrate that multiple stem cell lines can be propagated by laser-based passage and that the size of resulting colonies can be easily controlled by varying the input section size.

### 3.2. Improved Consistency of Stem Cell Cultures

**Figure 2:**
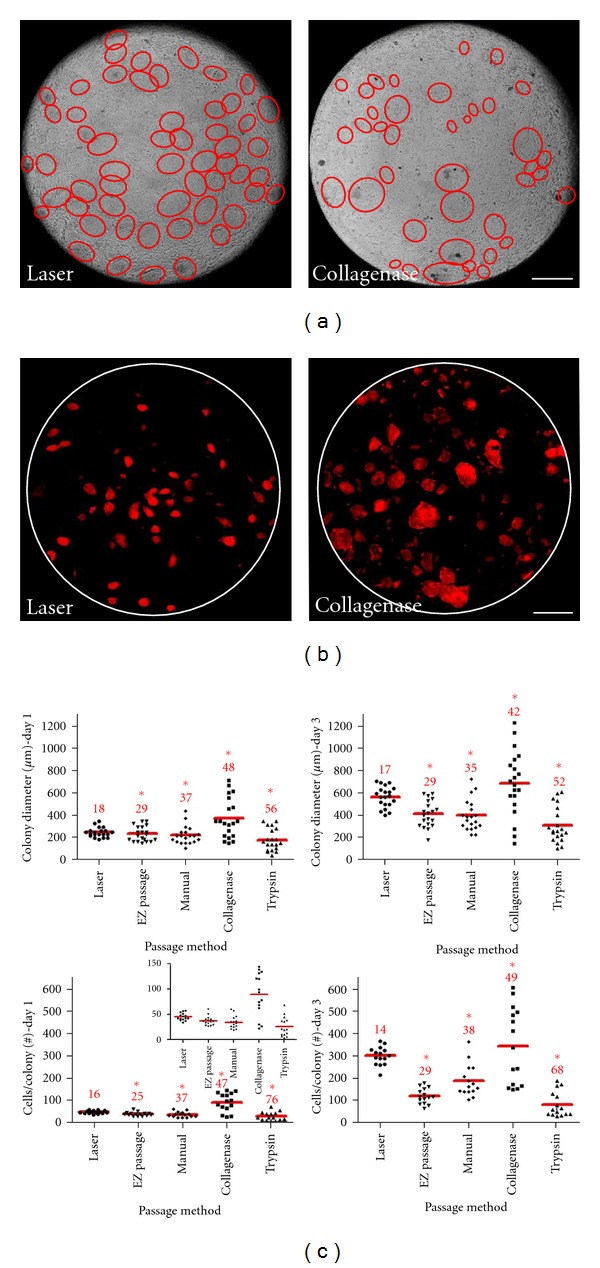
Improved uniformity of stem cell cultures by laser-mediated passage. (a) Brightfield images of iPSC cultures 2 days after laser-mediated passage (200 *μ*m sections, left) or collagenase passage (right). Colonies are shown by red outline. Scale bar, 1 mm. (b) Alkaline phosphatase (AP) staining of iPSC colonies 1 day after laser-mediated passage (200 *μ*m sections, left) or collagenase passage (right). Scale bar, 1 mm. (c) Colony size of iPSC cultures on days 1 and 3 after laser-mediated passage (200 *μ*m sections), StemPro EZPassage Disposable Stem Cell Passaging Tool (EZ Passage), manual passage using a pipette tip, collagenase treatment, or trypsin dissociation of cells. Longest diameter of each colony was manually measured using brightfield images (top, *n* = 20 colonies per data point). Number of cells per colony was manually counted using Hoechst stained cultures (bottom, *n* = 15 colonies per data point). Data are shown as scatter plot with red line indicating mean. The CV is shown in red text above each sample. Asterisks (*) indicate variances that are statistically significant when compared to laser-mediated passage using ANOVA, with a *P* ≤ 0.05 considered significant.

Laser-mediated passage was compared with traditional passaging techniques, both manual and enzymatic. iPSC cultures (BIMR 6) were passaged by (1) laser-mediated passage using 200 *μ*m sections, (2) manual passage using the StemPro EZPassage Disposable Stem Cell Passaging Tool (Invitrogen), (3) manual passage using a pipette tip (performed by an individual with 6-years experience), (4) enzymatic passage using collagenase, and (5) enzymatic passage using trypsin. Manual passage approaches generated significantly more uniform colonies than the enzymatic methods. Comparing laser-mediated passage with collagenase-based passage, image analysis of laser-passaged cultures revealed more homogeneous colony formation than collagenase passaged cultures (Figures 2(a) and 2(b). Stem cell cultures passaged by all methods were analyzed with respect to colony diameter and cells per colony (Figure 2(c)). Laser-mediated passage resulted in the most uniform colonies measuring 240 ± 43 *μ*m (18% CV) in diameter containing 45 ± 7 (16% CV) cells per colony one day after passage. Enzymatic passage by collagenase or trypsin resulted in significantly variable sized colonies measuring 365 ± 177 *μ*m (48% CV) and 172 ± 97 *μ*m (56% CV) in diameter containing 90 ± 42 (47% CV) and 25 ± 19 (76% CV) cells per colony, respectively. Manual passage techniques using a pipette tip or the EZPassage tool resulted in more similar sized colonies measuring 214 ± 9 *μ*m (37% CV) with 34 ± 3 (37% CV) cells per colony and 226 ±65 *μ*m (29% CV) in diameter with 37 ± 9 (25% CV) cells per colony, respectively. However, the EZPassage tool does not allow colonies growing at the edge of each well to be propagated, leaving >25% of the culture unsectioned (data not shown). Statistical analysis of variance showed that the stem cell cultures propagated by laser-mediated passage varied significantly less than (*P* value < 0.0001) cultures passaged manually or by enzymatic methods, demonstrating that laser-mediated passage results in more consistent stem cell cultures than all other methods. Comparable results were also obtained using the BIMR A iPSC line (data not shown).

### 3.3. Pluripotency, Quality, and Stability of Stem Cells after Laser-Mediated Passage

**Figure 3:**
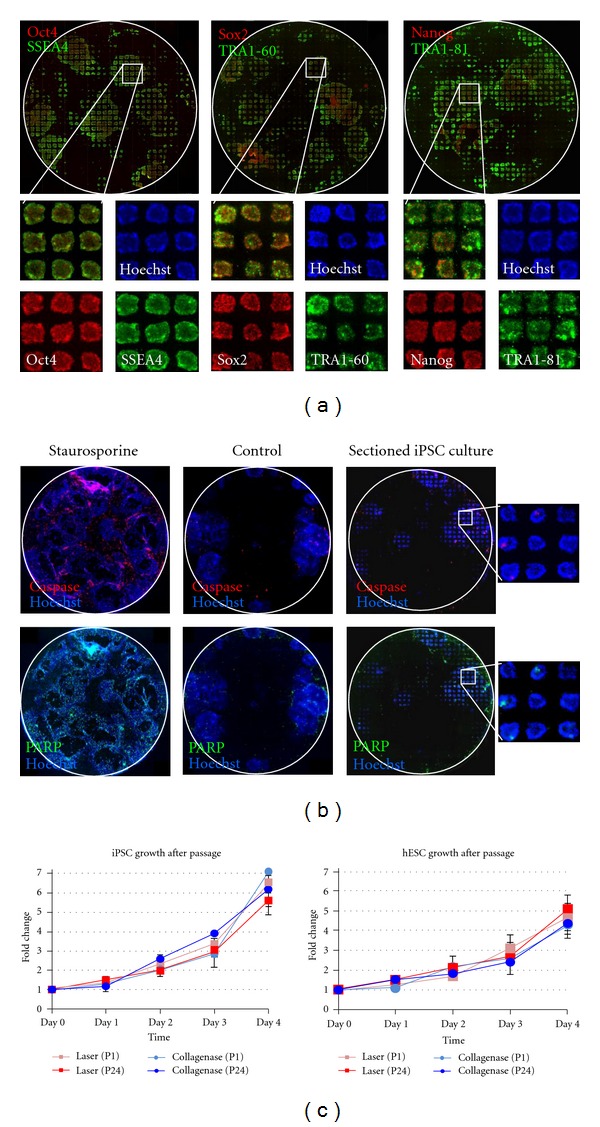
Quality of iPSC cultures after laser-mediated passage. (a) Immunocytochemical analysis of Oct4, Sox2, Nanog, SSEA4, TRA1-60, and TRA1-81 expression immediately following laser-mediated sectioning of iPSC cultures (BIMR L) into 200 *μ*m sections. Hoechst was used as a nuclear counterstain. Note that all markers are expressed homogeneously across iPSC clumps. Scale bar, 1 mm. (b) Immunocytochemical analysis of apoptosis markers, caspase-3, and cleaved PARP, following laser-mediated sectioning of iPSC cultures. Hoechst was used as a nuclear counterstain. Scale bar, 1 mm. (c) Analysis of iPSC (BIMR 6, left) and ESC (H9, right) growth following propagation using laser-mediated passage or collagenase passage. *P* indicates passage number. Data are shown as mean + s.d. (*n* = 3).

The effect of the laser on human iPSC and ESC quality and pluripotency was examined immediately following laser-mediated sectioning of stem cell cultures into 200 *μ*m sections. As shown in Figure 3(a), pluripotency markers such as Oct4, Sox2, Nanog, SSEA4, TRA1-60, and TRA1-81 were highly expressed in sectioned iPSC cultures. Image analysis demonstrated that all markers were expressed homogeneously across sections, even in cells right next to the laser sectioning lines. In addition, cells right next to the laser cutting lines did not show any significant increase in apoptosis, as measured by immunocytochemical analysis of activated caspase-3 and cleaved PARP four hours after laser-mediated sectioning (Figure 3(b)). Incubation of replicate cultures overnight (i.e., cultures were sectioned into 200 *μ*m sizes and then given fresh medium) resulted in significant growth of cells into the areas previously sectioned using the laser. Morphological and immunocytochemical analysis of these cultures (using the same pluripotency and apoptosis markers above) indicated that cells regrown into the laser sectioning area were indeed undifferentiated human iPSCs. The laser was then used to section a wider area (~1000 *μ*m) into cultures for analysis of growth over several days. Again, these cultures showed no change in morphology, apoptosis, and pluripotency marker expression, indicating that laser processing did not affect stem cell self-renewal or pluripotency (Supplemental Figure 1 of the supplementary material available online at doi:10.1155/2012/926463).

In addition, laser-mediated passage of human iPSCs and ESCs did not alter cell growth as the cells exhibited equivalent growth rates as compared with collagenase passaged cells after multiple rounds of expansion (Figure 3(c)).

**Figure 4:**
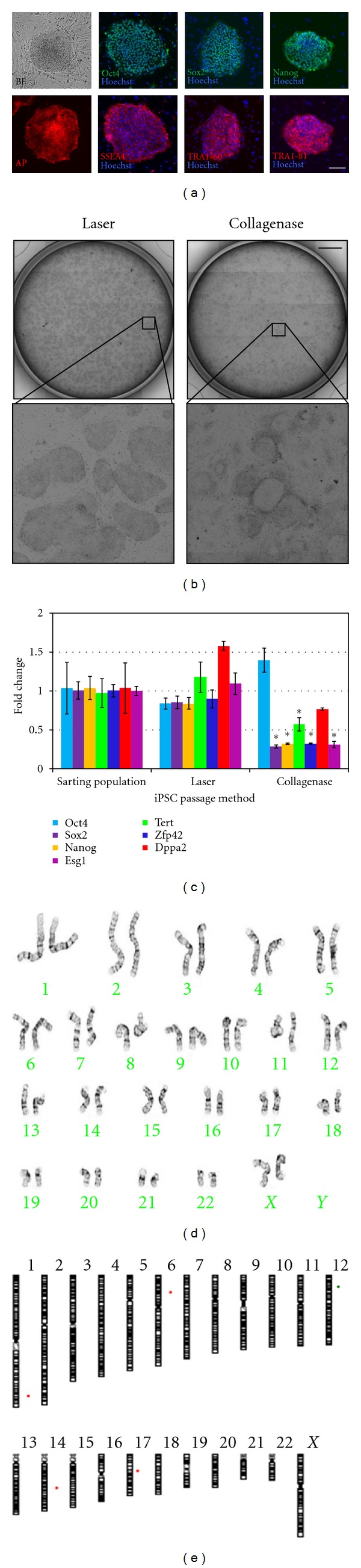
Pluripotency and stability of stem cells after laser-mediated passage. (a) Colony morphology (brightfield, BF) and immunocytochemical analysis of Oct4, Sox2, Nanog, alkaline phosphatase (AP), SSEA4, TRA1-60, and TRA1-81 expression in human ESCs (H9) after 24 consecutive laser-mediated passages. Hoechst was used as a nuclear counterstain. Scale bar, 250 *μ*m. (b) Whole well brightfield images of human iPSC (BIMR 6) cultures after 10 consecutive laser-mediated passages or collagenase passages. Scale bar, 5 mm. (c) QRT-PCR analysis of stem-cell-associated gene expression in iPSCs (BIMR 6) after 10 consecutive laser-mediated passages or collagenase passages. The asterisks (*) indicate values that are statistically significant compared with the starting population of iPSCs. The data are presented as mean ± s.d. (*n* = 3). Statistical analysis was performed using *t*-test, with a *P* value ≤ 0.05 considered to be significant. (d) Normal karyotype of H9 human ESCs after 24 laser-mediated passages (6 months). (e) Schematic depicting genomic abnormalities of H9 ESCs at P35 (starting population) and at P59, after 24 laser-mediated passages (6 months), as determined by aCGH. No new subkaryotypic abnormalities were detected after 24 passages. Red bars indicate a deletion. Green bars indicate an amplification. See Table 1 for complete list of aberrations found in these cells.

To further assess the potential laser effects on human iPSC and ESC stability and pluripotency, stem cell cultures were propagated using laser-mediated passage over long-term culture (two iPSC lines were maintained for >5 passages (5 weeks), one iPSC line was maintained for >10 passages (2.5 months), and BIMR 6 iPSCs and H9 ESCs were maintained for >24 passages (>6 months)) and compared with replicate cultures passaged using collagenase. Image analyses of cultures propagated by laser-mediated passage showed no change in morphology with all cells exhibiting a high nuclear to cytoplasmic ratio typical of pluripotent stem cells (Figure 4(a)). Immunocytochemical analyses of these cultures demonstrated iPSCs and ESCs continued to express characteristic pluripotency markers including alkaline phosphatase (AP), Oct4, Sox2, Nanog, SSEA4, TRA1-60, and TRA1-81 (Figure 4(a)).

Visual comparison showed that human BIMR 6 iPSC cultures propagated using laser-mediated passage were of higher quality over time than those propagated using collagenase. Cultures propagated by laser-mediated passage contained many more undifferentiated, compact stem cell colonies with clear discernible colony borders compared to cultures propagated using collagenase. Collagenase passaged cultures had a greater tendency for spontaneous differentiation (Figure 4(b)). To quantify these phenotypic observations, QRT-PCR analyses were performed using known markers for undifferentiated stem cells. As shown in Figure 4(c), iPSCs propagated using laser-mediated passage continued to express high levels of stem cell-associated genes, including *Oct4* (*Pou5f1*), *Sox2*, *Nanog*, *Tert*, *Zfp42* (*Rex1*), *Dppa2*, and *Esg1 *(*Dppa5*), similar to the starting population of iPSCs (no statistical difference, *P* value ≥ 0.1). In contrast, iPSCs propagated using collagenase resulted in a significant decrease in expression of *Sox2*, *Nanog*, *Tert*, *Zfp42*, and *Esg1* when compared to the starting population of iPSCs (*P* value < 0.05, Figure 4(c)). Comparison of human ESC cultures passaged by both methods did not show any significant morphological changes or differences in gene expression (Supplemental Figure 2), although human ESC cultures were more established, later passage cultures than human iPSC cultures used in these experiments.

Data from multiple groups have shown that H9 human ESCs maintain a stable karyotype over long-term culture [1, 17, 22]. These cells were therefore used to examine the genetic stability of stem cells after long-term propagation using laser-mediated passage. Karyotype analysis of H9 ESCs after 24 consecutive laser-mediated passages showed no change in karyotype with all cells having a normal diploid karyotype (6 months, Figure 4(d)). To detect subkaryotypic alterations, array comparative genomic hybridization (aCGH) was also performed using the Stemarray (Figure 4(e), Table 1). No subkaryotypic alterations were detected in human ESCs propagated for 24 consecutive passages (P59) by laser-based passaging relative to the starting population (H9, P35), suggesting that the genome of laser-mediated passaged cells is both normal and stable. It is important to note that both subkaryotypic and karyotypic alterations were observed in H9 ESCs after consecutive passaging by collagenase for 4 and 6 months, respectively (data not shown). In addition, no subkaryotypic changes were detected in human iPSCs (BIMR 6) propagated for 10 consecutive passages by laser-mediated passage relative to the starting population (data not shown). Although genetic stability of iPSCs was only analyzed after 2.5 months, taken together with 6-month human ESC results, these data suggest that the genome of laser-mediated passaged stem cells is and stable.

### 3.4. Improved Differentiation Potential of EBs Generated after Laser-Mediated Passage

**Figure 5:**
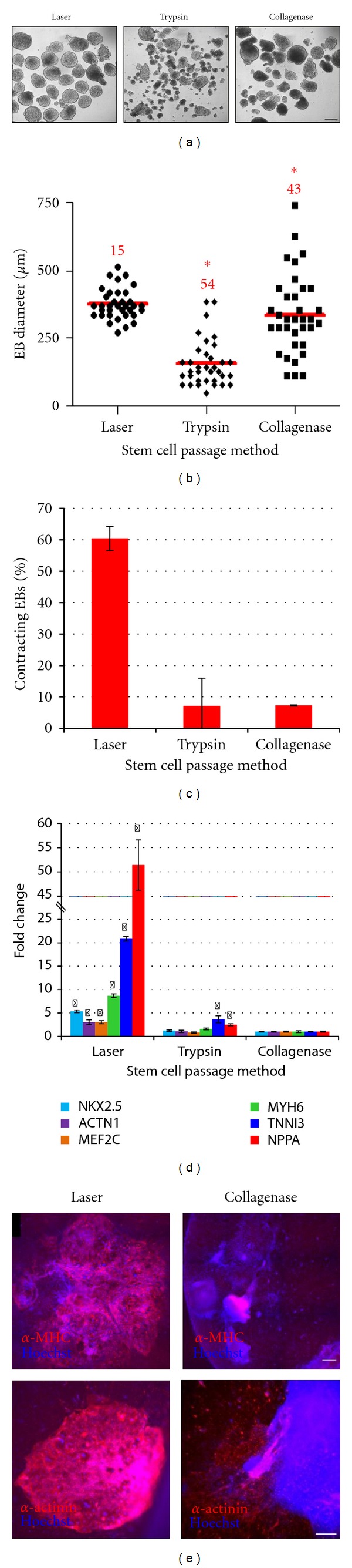
iPSCs propagated by laser-mediated passage differentiated more efficiently into cardiomyocytes. (a) Brightfield image of day 4 EBs generated from iPSC cultures (BIMR A) propagated by laser-mediated passage, trypsin dissociation, or collagenase treatment. Scale bar, 250 *μ*m. (b) Size of EBs generated from iPSC cultures propagated by laser-mediated passage, trypsin dissociation, or collagenase treatment (*n* = 35 EBs per data point). Data are shown as scatter plot with red line indicating mean and CV shown in red text above each sample. The asterisks (*) indicate variances that are statistically significant when compared to laser using ANOVA, with *P* ≤ 0.05 considered significant. (c) Percentage of EBs containing contracting areas. Data are shown as mean + s.d. (*n* = 2 independent experiments containing 75 EBs/sample in each experiment). (d) QRT-PCR analysis of cardiomyocyte-associated gene expression in EBs generated using iPSC cultures propagated by laser-mediated passage, trypsin dissociation, or collagenase treatment. The asterisks (*) indicate values that are statistically significant as compared with EBs generated from collagenase passaged iPSC cultures. The data are presented as mean ± s.d. (*n* = 3). Statistical analysis was performed using *t*-test with *P* ≤ 0.05 considered significant. (e) Expression of cardiomyocyte markers, *α*-MHC and *α*-actinin, in EBs generated from iPSC cultures propagated by laser-mediated passage or collagenase treatment on day 22 of cardiac differentiation. Hoechst was used as a nuclear counterstain. Scale bars, 250 *μ*m.

To test the differentiation potential of these cells, in vitro differentiation assays of human iPSCs were performed after propagation using laser-mediated passage (160 *μ*m sections) or enzymatic passage. iPSCs passaged by either methodology efficiently formed well-defined embryoid bodies in suspension culture which could differentiate into derivatives of all three primary germ layers including endodermal cells (Sox17, Afp), mesodermal cells/cardiac muscle cells (brachyury, *α*-MHC), and ectodermal cells/neurons (Nestin, Map2, Supplemental Figure 3). Morphological analysis of the resulting EB populations showed that EBs generated from laser-passaged iPSCs were more uniform in size than those generated from enzymatically passaged iPSCs (Figure 5(a)). To quantify these observations, the diameter of resulting EB populations was measured manually using images acquired on day 4 of suspension culture. As shown in Figure 5(b), laser-mediated passage resulted in significantly more uniform EBs (374 ± 56 *μ*m; 15% CV) than enzymatic passage by either collagenase (336 ± 145 *μ*m; 43% CV) or trypsin (158 ± 85 *μ*m; 54% CV). Statistical analysis of variance showed that EBs generated using stem cell cultures propagated by laser-mediated passage were significantly more uniform (*P* value < 0.0001) than EBs generated using enzymatically passaged cultures, demonstrating that laser-mediated passage results in more consistent EB cultures than other methods.

Several studies have shown that heterogeneity in human ESC colony size and resulting EB aggregate size results in variability in differentiation experiments and significant decreases in differentiation yields [23–26]. The effect of EB homogeneity on differentiation potential of human iPSCs into cardiomyocytes was examined. EBs were generated using iPSC colonies formed 5 days after laser-mediated passage (160 *μ*m sections) or enzymatic passage. All EBs were differentiated using a standard multistage protocol, growing EBs in suspension culture for 4 days followed by adherent cell culture for an additional 18 days [27]. Cardiomyocyte differentiation potential was analyzed on day 22 of differentiation by manual counting of contracting EBs. EBs produced using enzymatically passaged iPSCs yielded a small proportion of beating EBs (~7%), whereas laser-mediated passaged iPSCs resulted in a significantly higher proportion (~60%) of contracting EBs (Figure 5(c)). QRT-PCR analyses confirmed these results with EBs generated from laser-mediated passaged cultures showing 3- to 51-fold higher expression of cardiomyocyte genes, *Nkx2.5*, *Actn1 *(*α-actinin*), *Mef2C*, *Myh6* (*α-Mhc*), *TnnI3*, and *NppA* (*Anp*), than EBs generated from collagenase passaged cultures (Figure 5(d)). Similarly, immunocytochemical analyses demonstrated that EBs produced from laser-mediated passaged iPSCs contained substantially more cardiac cells within each EB (i.e., EBs contained more cells staining positive for known cardiomyocyte markers) than EBs produced from collagenase passaged cells (*α*-MHC and *α*-actinin shown in Figure 5(e)). EBs generated from all populations stained positive for all markers tested including *α*-MHC, *α*-actinin, cardiac troponin, and NPPA (data not shown).

To further analyze the effect of EB homogeneity on differentiation potential, EBs generated from iPSC colonies formed 5 days after laser-mediated passage (160 *μ*m sections) or enzymatic passage were differentiated into neural rosettes using a modified multistep protocol. Ability to differentiate into neural rosettes was analyzed on day 11 of differentiation by manual counting of EBs containing ≥1 neural rosette. EBs produced using laser-mediated passaged iPSCs resulted in 95% of EBs containing neural rosettes, while EBs generated by trypsin or collagenase passaged iPSCs yielded only 29% and 32% of EBs containing neural rosettes, respectively (data not shown). These data indicate increased homogeneity in human iPSC colonies and resultant EBs result in significant increases in differentiation yield of iPSCs.

To investigate the effect of EB size on differentiation potential of human iPSCs were examined. iPSCs were propagated by laser-mediated passage at varying section sizes (80, 160, and 240 *μ*m sections). Five days after passage, EBs were generated and differentiated into cardiomyocytes as described above. Homogeneous EB populations (≤15% CVs) of varying sizes, 278, 418, and 528 *μ*m in diameter, were produced from 80, 160, and 240 *μ*m section sizes, respectively (Supplemental Figure 4). Analysis of cardiomyocyte differentiation potential showed that 55% of EBs generated from 160 *μ*m sections were contracting, while only 38% and 21% of EBs generated from 240 *μ*m and 80 *μ*m sections were contracting. Taken together, these data indicate that increased homogeneity in human iPSC colonies and resultant EBs, as well as EB size, significantly increase the differentiation yield of iPSCs. The ability to reproducibly generate uniform, size-specific colonies which subsequently result in more uniform, size-specific EB populations decreases variability in differentiation experiments and enhances differentiation yields of both ESC and iPSCs into specialized cell types.

## 4. Discussion

The lack of standardization in passage techniques for stem cell derivation and propagation is a major limitation within the stem cell field. Because universal protocols for human stem cell cultures have not been adopted, it is currently difficult to compare and interpret scientific data from cells cultured in different conditions. Passage method differences have significantly confounded the understanding of intra- and interline differences in gene expression data, expression of stem cell- and lineage-associated markers, miRNA signatures, and epigenetic profiles [28–31]. Although human ESC lines have distinct genotypes, it is unlikely that reported differences in cell lines (e.g., up to 65% variation in gene expression data across two ESC lines) can be attributed to genetic variation alone, as <2% variation in gene expression has been found in adult human tissues of different individuals [28, 32, 33]. Likewise, discrepancies associated with differentiation protocols and reported differentiation capabilities and efficiencies of stem cells into specialized cell types may be due to the lack of standardization [34]. Adoption of standardized protocols should greatly improve determination of the role of inherent genetic variation, environmental niche, and/or procedural effects on stem cell quality, self-renewal, pluripotency, and differentiation potential.

Laser-mediated passage provides a novel method for expansion of human ESCs/iPSCs which can be used to create standardized, traceable procedures for the production of GMP-quality stem cell lines without requirement for enzymes. This method combines the benefits of both manual and enzymatic passage techniques, allowing efficient, automated passaging of undifferentiated stem cell cultures into uniform-sized stem cell sections within a sterile closed environment. Laser-mediated passage is compatible with a variety of culture methods including animal-free, feeder-free-based conditions, and serum-free defined media conditions. Notably, this approach is not susceptible to inter-individual variation reducing the need for skilled technicians to create high-quality stem cell cultures.

Laser-mediated passage does not involve the use of enzymes and therefore should better maintain the genetic stability of human ESCs and iPSCs in long-term culture (3–12 months, [10–14]). The results show that H9 ESCs maintained a stable karyotype over six months (>24 passages). More importantly, laser-mediated passage did not induce subkaryotypic alterations over time in H9 ESCs (6 months, and iPSCs (2.5 months)) as monitored by aCGH. The more sensitive aCGH data suggests that laser-mediated passage maintains genetic integrity of human ESCs/iPSCs. Importantly, genetic abnormalities were detected in H9 ESCs after consecutive passaging by collagenase during the same time period. Results also showed that human iPSCs and ESCs propagated using laser-mediated passage maintained a normal stem cell morphology and continued to express high levels of stem-cell-associated genes and proteins. Although teratoma analyses were not performed on these cells, in vitro differentiation analyses of laser-mediated passaged iPSCs demonstrated the cells could spontaneously differentiate into derivatives of all three primary germ layers and could differentiate into cardiomyocytes and neural rosettes. In addition, iPSCs propagated by laser-mediated passage have been differentiated into motor neurons, RPE cells, endoderm progenitors, and hepatocytes-like cells (data not shown); taken together these data indicate that laser-mediated passage does not affect stem cell pluripotency. Likewise, laser-mediated passage did not alter the growth rate of stem cells or increase expression of apoptotic markers, all supporting that the laser sectioning did not affect stem cell quality, self-renewal, or pluripotency.

Laser-mediated passage provides control of stem cell colony size. Regular passage schedules can be established by selection of section size. A section size of ~200 *μ*m has enabled routine splitting of all ESC/iPSC lines every 7 days, allowing for more efficient planning of experiments. An overall passage efficiency of 85% combined with more uniform section sizes (20% CV), enables a larger proportion of ESC/iPSC colonies to contribute to culture expansion reducing the number of plates required for culture maintenance. Compatibility with conventional robotic systems enables scalability of culture needs. Additionally, the ability to control input section size, particularly smaller sizes, allows more effective creation of stem cell colonies in multi-well plates for large-scale experimentation and screening purposes. It is also likely that stem cell section size will affect cryopreservation and genetic modification efficiency of ESCs and iPSCs [35–38].

Laser-mediated passage involves sectioning the entire well systematically without respect for the boundaries of the colonies. Well-established undifferentiated stem cell cultures are easily propagated using this technique. For newly derived ESC/iPSC cultures, early passage ESC/iPSC lines, or less stable lines, which tend to have more spontaneous differentiation, a combination of manual selection or laser purification of colonies followed by laser-mediated passage would be recommended. One of the more important results of this approach showed that over time, stem cell cultures (in particular, early passage iPSCs which tend to be more susceptible to differentiation than later passage, more established ESCs) are of higher quality than those maintained by collagenase treatment. It is likely that passage of homogeneous sections is important for maintaining undifferentiated stem cells and limiting the differentiation of colonies. Therefore, potentially early passage ESCs/iPSCs will require less colony isolation before expansion using laser-mediated passage.

One of the more important outcomes of this study showed that uniform human iPSC colonies produced after laser-mediated passage resulted in a more homogeneous population of EBs, with respect to size and shape, with greater differentiation efficiency as compared with typical EB cultures derived from enzyme passaged cultures. EBs generated from laser-mediated passaged iPSCs resulted in a significant increase in cardiomyocyte yield, with up to 8.5-fold greater beating incidence than EBs generated from collagenase passaged iPSCs. The ability to reproducibly generate uniform colonies using laser-mediated passage resulting in EBs that are more uniform in size and shape will decrease variability in differentiation experiments and enhance differentiation yields of both ESC and iPSCs into specialized cell types. These yield enhancements could significantly reduce the cost of stem cell experimentation both in terms of labor and materials. Potentially, uniform colony formation will also augment differentiation yields of stem cells when performing direct differentiation procedures (i.e., without an EB intermediate).

## 5. Conclusions

In conclusion, proper maintenance of human stem cells is essential for successful utilization of ESCs and iPSCs as tools in developmental and drug discovery studies and in regenerative medicine. Standardization is critical for all future applications of stem cells and necessary in order to fully understand the potential of these cells and the differences observed among varying stem cell lines and between ESCs and iPSCs. Laser-mediated passage is an innovative method for maintenance and expansion of stem cell lines, without introducing genetic instability, which is generically applicable to all cell lines and to all technicians regardless of skill. This approach provides an efficient, standardized protocol for the propagation of human ESCs and iPSCs, which should significantly reduce the inconsistency and variability within the stem cell field. Laser-mediated passage allows for traceability and ensures reproducible production of stem cell lines according to standard operating procedures, all of which are necessary to manufacture stem cells for use in clinical/therapeutic applications.

##  Author's Contribution

C. Peterson and A. Soundararajan contributed equally to the paper.

## Supplementary Material

Supplemental Figure 1: The effect of the laser on human iPSC pluripotency was examined following laser-mediated sectioning of stem cell cultures. Cultures were cut into 1000 um wide areas by the laser and examined on day 0, 1, and 3 for expression of pluripotency markers. Analysis of these cultures on days 1 and 3 showed that iPSCs had grown into the areas previously cut by the laser. These cells showed no change in morphology or marker expression, indicating that laser processing did not affect stem cell self-renewal or pluripotency.
Supplemental Figure 2: Expression of stem cell-associated genes in human ESCs following laser-mediated passage or collagenase passage was analyzed by QRT-PCR. Human ESC cultures passaged by both methods did not show any significant differences in gene expression. All cells expressed high levels of *Oct4 (Pou5f1)*, *Sox2*, *Nanog*, *Tert*, *Zfp42 (Rex1)*, *Dppa2*, and *Esg1 (Dppa5)*.
Supplemental Figure 3: The differentiation potential of human iPSCs propagated by laser-mediated passage was analyzed by in vitro differentiation assays. These cells formed well-defined EBs in suspension culture which could differentiate into derivatives of all three primary germ layers including endodermal cells (Sox17, Afp), mesodermal cells/cardiac muscle cells (brachyury, *α*-MHC), and ectodermal cells/neurons (Nestin, Map2).
Supplemental Figure 4: The effect of EB size on differentiation potential of human iPSCs was examined using iPSCs propagated by laser-mediated passage at varying section sizes. Five days after passage, EBs were generated from each section size and differentiated into cardiomyocytes. Homogeneous EB populations of varying sizes, 278, 418, and 528 *μ*m in diameter, were produced from 80, 160, and 240 *μ*m section sizes, respectively (Supplemental Figure 4a, 4b). Analysis of cardiomyocyte differentiation potential showed that 55% of EBs generated from 160 *μ*m sections were contracting, while only 38% and 21% of EBs generated from 240 *μ*m and 80 *μ*m sections were contracting (Supplemental Figure 4c).

## Figures and Tables

**Table 1:** Regions in H9 human ESCs with genomic abberations as determined by aCGH (corresponding schematic is shown in Figure 4(e)). No subkaryotypic alterations were detected in hESCs propagated for 24 consecutive passages (H9 P59) relative to the starting hESC population (H9 P35), suggesting that the genome of laser-mediated passaged cells is both normal and stable. Data is reported using genome built HG18. log_2_⁡ ratios ≥ 0.6 are amplifications (amp) or ≤−1.0 are deletions (del) found in all cells. log_2_⁡ ratios < 0.6 or >−1.0 represent mosaicism within the culture.

Chromosome: region	Cytoband	Size (Mb)	# Probes	Amp/Del	H9 P35 log⁡_2_⁡ ratio	H9 P59 log⁡_2_⁡ ratio	Annotations
Chr1: 224141493-224195678	q42.12	0.054	14	Amp	0.528	0.632	LEFTY1, PYCR2, LEFTY2
Chr6: 31663619-31691605	p21.33	0.028	3	Amp	0.686	1.220	LST1, NCR3, AIF1
Chr12: 21580165-22105263	q12.1	0.282	12	Del	−0.495	−0.480	GYS2, LDHB, KCNJ8, ABCC9, CMAS
Chr14: 62486603-62852257	q23.2	0.366	7	Amp	0.560	0.432	KCNH5, RHOJ, GPHB5
Chr17: 35097815-35153082	q12	0.055	15	Amp	0.469	0.485	PERLD1, ERBB2, C17orf37, GRB7
