# Supplementary material for: Laser-Based Propagation of Human iPS and ES Cells Generates Reproducible Cultures with Enhanced Differentiation Potential
Source: Stem Cells Int. 2012 May 30;2012:926463. doi: 10.1155/2012/926463 (PMC3369526; doi:10.1155/2012/926463)
Supplement: Supplementary file 1 — Supplemental Figure 1: The effect of the laser on human iPSC pluripotency was examined following laser-mediated sectioning of stem cell cultures. Cultures were cut into 1000 um wide areas by the laser and examined on day 0, 1, and 3 for expression of pluripotency markers. Analysis of these cultures on days 1 and 3 showed that iPSCs had grown into the areas previously cut by the laser. These cells showed no change in morphology or marker expression, indicating that laser processing did not affect stem cell self-renewal or pluripotency. Supplemental Figure 2: Expression of stem cell-associated genes in human ESCs following laser-mediated passage or collagenase passage was analyzed by QRT-PCR. Human ESC cultures passaged by both methods did not show any significant differences in gene expression. All cells expressed high levels of Oct4 (Pou5f1), Sox2, Nanog, Tert, Zfp42 (Rex1), Dppa2, and Esg1 (Dppa5). Supplemental Figure 3: The differentiation potential of human iPSCs propagated by laser-mediated passage was analyzed by in vitro differentiation assays. These cells formed well-defined EBs in suspension culture which could differentiate into derivatives of all three primary germ layers including endodermal cells (Sox17, Afp), mesodermal cells/cardiac muscle cells (brachyury, α-MHC), and ectodermal cells/neurons (Nestin, Map2). Supplemental Figure 4: The effect of EB size on differentiation potential of human iPSCs was examined using iPSCs propagated by laser-mediated passage at varying section sizes. Five days after passage, EBs were generated from each section size and differentiated into cardiomyocytes. Homogeneous EB populations of varying sizes, 278, 418, and 528 μm in diameter, were produced from 80, 160, and 240 μm section sizes, respectively (Supplemental Figure 4a, 4b). Analysis of cardiomyocyte differentiation potential showed that 55% of EBs generated from 160 μm sections were contracting, while only 38% and 21% of EBs generated from 240 μm and 80 μm sections [file 926463.f1.docx]

Supplemental Figure 1.
