# Supplementary figures and images for: Laser-Based Propagation of Human iPS and ES Cells Generates Reproducible Cultures with Enhanced Differentiation Potential
Source: Stem Cells Int. 2012 May 30;2012:926463. doi: 10.1155/2012/926463 (PMC3369526; doi:10.1155/2012/926463)

Supplemental Figure 2.


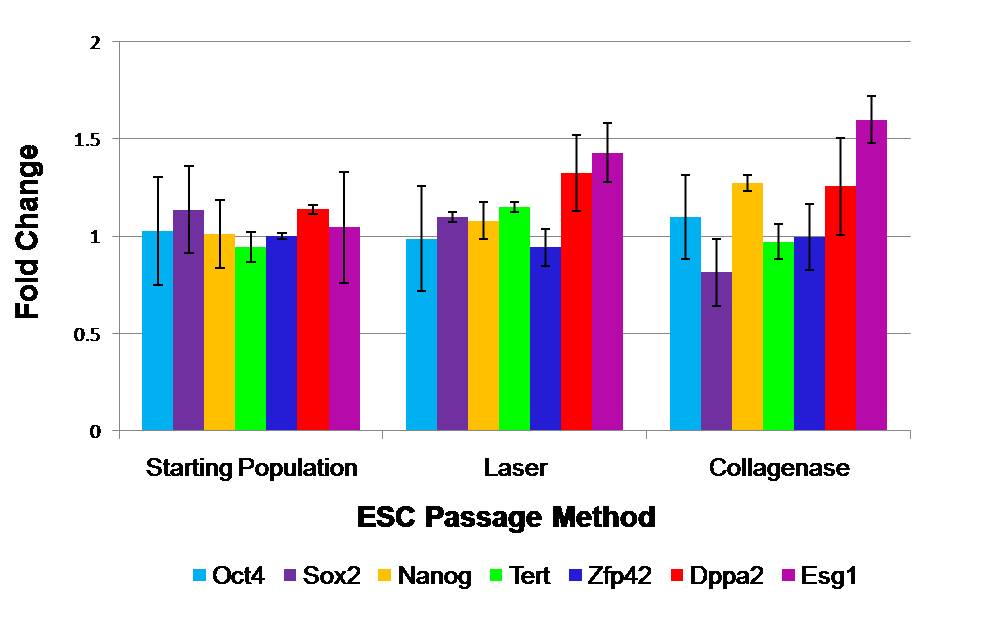

Supplement: Supplementary file 2 [file 926463.f2.docx]

Supplemental Figure 3.


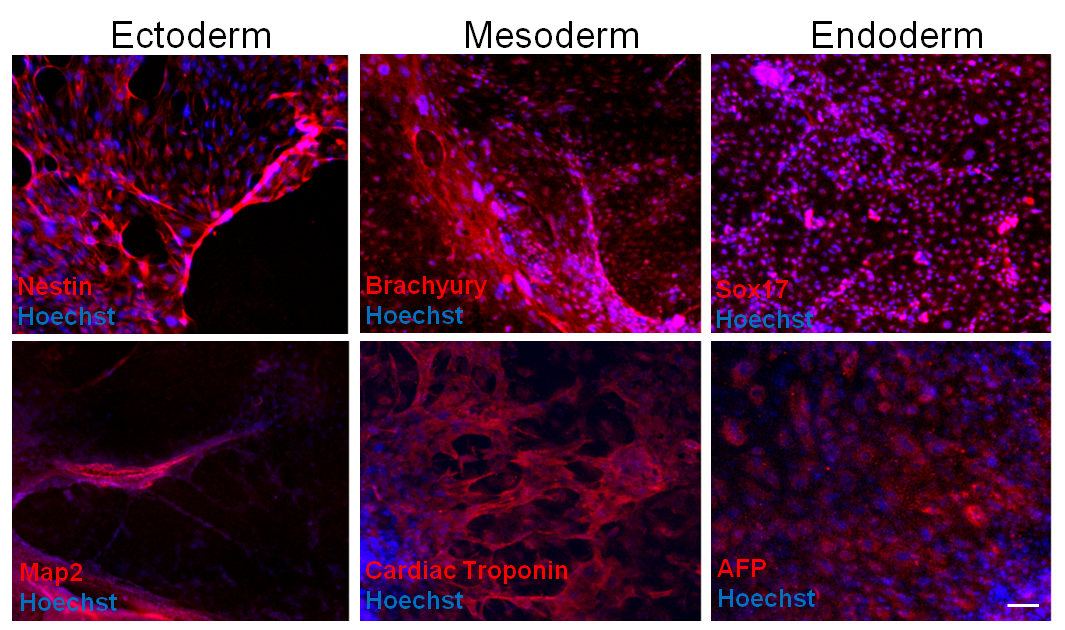

Supplement: Supplementary file 3 [file 926463.f3.docx]

Supplemental Figure 4a.


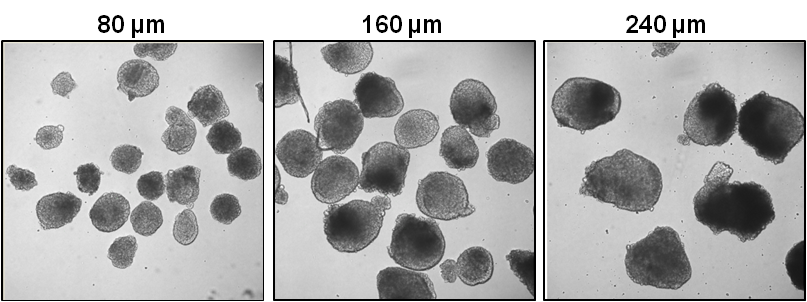


Supplemental Figure 4b.

Supplemental Figure 4c.

Supplement: Supplementary file 4 [file 926463.f4.docx]
